# Safety in treatment: Classical pharmacotherapeutics and new avenues for addressing maternal depression and anxiety during pregnancy

**DOI:** 10.1016/j.pharmr.2025.100046

**Published:** 2025-02-10

**Authors:** Merel Dagher, Catherine M. Cahill, Anne M. Andrews

**Affiliations:** 1 Department of Psychiatry and Biobehavioral Sciences, Hatos Center for Neuropharmacology, Semel Institute for Neuroscience and Human Behavior, University of California Los Angeles, Los Angeles, California; 2 Neuroscience Interdepartmental Program, University of California Los Angeles, Los Angeles, California; 3 Department of Chemistry and Biochemistry, University of California, Los Angeles, Los Angeles, California; 4 California Nanosystems Institute, University of California, Los Angeles, Los Angeles, California

## Abstract

We aimed to review clinical research on the safety profiles of antidepressant drugs and associations with maternal depression and neonatal outcomes. We focused on neuroendocrine changes during pregnancy and their effects on antidepressant pharmacokinetics. Pregnancy-induced alterations in drug disposition and metabolism impacting mothers and their fetuses are discussed. We considered evidence for the risks of antidepressant use during pregnancy. Teratogenicity associated with ongoing treatment, new prescriptions during pregnancy, or pausing medication while pregnant was examined. The Food and Drug Administration advises caution regarding prenatal exposure to most drugs, including antidepressants, largely owing to a dearth of safety studies caused by the common exclusion of pregnant individuals in clinical trials. We contrasted findings on antidepressant use with the lack of treatment where detrimental effects to mothers and children are well researched. Overall, drug classes such as selective serotonin reuptake inhibitors and serotonin norepinephrine reuptake inhibitors appear to have limited adverse effects on fetal health and child development. In the face of an increasing prevalence of major mood and anxiety disorders, we assert that individuals should be counseled before and during pregnancy about the risks and benefits of antidepressant treatment given that withholding treatment has possible negative outcomes. Moreover, newer therapeutics, such as ketamine and *κ*-opioid receptor antagonists, warrant further investigation for use during pregnancy.

**Significance Statement:**

The safety of antidepressant use during pregnancy remains controversial owing to an incomplete understanding of how drug exposure affects fetal development, brain maturation, and behavior in offspring. This leaves pregnant people especially vulnerable, as pregnancy can be a highly stressful experience for many individuals, with stress being the biggest known risk factor for developing a mood or anxiety disorder. This review focuses on perinatal pharmacotherapy for treating mood and anxiety disorders, highlighting the current knowledge and gaps in our understanding of consequences of treatment.

## I Introduction: safety of using antidepressants and anxiolytics during pregnancy

According to the Centers for Disease Control, 1 in 7 women of reproductive age is prescribed medication for anxiety or mood disorders (Dawson et al, 2016). Estimates of maternal perinatal depression or anxiety range from 5% to 25% and are likely underestimates owing to the ongoing social stigma associated with mental health conditions (Corrigan and Watson, 2002). Moreover, ∼15% of women experience a new episode of depression within the first 3 months postpartum (Woody et al, 2017). Psychopathology associated with postpartum depression has detrimental outcomes where suicide accounts for 20% of maternal postpartum deaths (Lindahl et al, 2005).

Approximately 5%–8% of pregnant women with mood or anxiety disorders will initiate antidepressant treatment during pregnancy (Andrade et al, 2008; Hanley and Mintzes, 2014). Approximately 4%–10% of pregnant women will continue taking antidepressants, with these numbers mainly accounting for selective serotonin reuptake inhibitors (SSRIs) (Wikman et al, 2020). While being prescribed or filling a prescription for an antidepressant is often used as a proxy for SSRI exposure, factors such as compliance (actually taking the medication), the severity of depressive symptomology, and the time frame of use during pregnancy are not always reliably reported (Palmsten and Hernández-Díaz, 2012). The safety of antidepressant use during pregnancy remains controversial due to an incomplete understanding of how drug exposure affects fetal development and brain maturation and behavior in offspring (Payne and Meltzer-Brody, 2009; Byatt et al, 2013). Moreover, social and cultural stigma are barriers to receiving treatment, particularly because most physicians advise limiting exposure to any substances during pregnancy (Osborne and Payne, 2015).

The Food and Drug Administration (FDA) website makes the following statement: “There are no adequate and well-controlled studies of SSRIs in pregnant women. At this time, FDA advises health care professionals not to alter their current clinical practice of treating depression during pregnancy. Healthcare professionals should report any adverse events involving SSRIs to the FDA MedWatch Program” (FDA, 2018). In contrast, lack of treatment of maternal mood and anxiety disorders are known to be associated with adverse health outcomes for offspring (Coussons-Read, 2013). A balanced understanding of the safety of antidepressants with respect to maternal health, fetal outcomes, and their interactions will improve clinical guidelines and recommendations. Additionally, knowledge about antidepressant safety during pregnancy will promote maternal autonomy by informing individual decisions to continue (or discontinue) pharmacotherapy during pregnancy.

Mild-to-moderate mood and anxiety disorders are treated with nonmedication therapies such as cognitive-behavioral therapy (Hofmann et al, 2012). Patients report the highest positive outcomes when “talk therapy” is used in combination with medication (Misri et al, 2010). Thus, tapering medication treatment while continuing or initiating behavioral interventions may be an effective option for treating mood and anxiety disorders during pregnancy that avoids exposing fetuses to the possible teratogenic effects of antidepressants. Yet, for many, particularly individuals with severe mood and anxiety disorders, behavioral therapies do not provide sufficiently effective treatment. Moreover, availability or accessibility of behavioral therapies are often limited owing to high out-of-pocket costs and lack of access to behavioral specialists, thus disadvantaging women with lower socioeconomic status and inadequate insurance coverage (Smith et al, 2009; Ross et al, 2019; Lee-Carbon et al, 2022). According to an international review on clinical practice guidelines regarding perinatal use of antidepressants, guidelines converge such that mild-to-moderate depression during pregnancy should be treated first with psychotherapy, before moving to pharmacotherapy (Molenaar et al, 2018). Integrated approaches using pharmacologic and behavioral therapies are warranted, particularly for more severe or refractory perinatal mood and anxiety disorders in light of evidence showing the efficacy of combined treatments (Cauli et al, 2019).

This review aimed to focus on perinatal pharmacotherapy for treating mood and anxiety disorders, and particularly, on 3 broad medication categories—SSRIs, serotonin norepinephrine reuptake inhibitors (SNRIs), and atypical antidepressants. Readers are directed to a previous systematic review that also discusses other psychotropics including benzodiazepine use during pregnancy on neonatal and childhood outcomes that include preclinical data (Creeley and Denton, 2019). In this review, we also discuss the prevalence of antidepressant use, pharmacology, and potential side effects for mothers and their offspring. We highlight pregnancy-induced changes in neuroendocrine systems and effects on drug pharmacokinetics that impact pregnant individuals and neonatal exposures. Additionally, new therapeutic agents are discussed that target serotonin (5HT)2A, *N*-methyl-d-aspartate (NMDA), and *κ* opioid receptors (KORs), for example.

The epidemiologic and clinical studies presented primarily include cisgender women in their datasets. We use the terms “pregnant people/individuals/persons” aiming to encompass all individuals who may become pregnant.

### A Materials and methods

A literature search strategy using PubMed and Google Scholar was used. Search terms included the following: antidepressant use during pregnancy, maternal mood and anxiety disorders, maternal depression, neonatal health, and children health outcomes with prenatal antidepressant exposure. Preclinical studies were excluded from the search to narrow the scope to human clinical research studies. Only a brief section on available preclinical models to investigate depressive-like and anxiety-like behaviors in rodents was included for context. Clinical studies were reviewed and evaluated for their study designs, study outcomes, and limitations. Only studies from peer-reviewed journals were included. The scope of this review included classical antidepressants that primarily target the monoamine systems, specifically serotonin, either as indirect agonists at monomamine transporters (eg, SSRIs) or enzyme inhibitors (eg, monoamine oxidase inhibitors [MAOIs]). We also examined therapies being developed as novel antidepressant drugs, especially for treatment-resistant depression (TRD). We specifically conducted literature searches on psychedelics and KOR antagonists, as these drug are being actively researched as novel antidepressants. We did not include augmentation medications in our review.

### B The Diagnostic and Statistical Manual of Mental Disorders criteria and prevalence of mood and anxiety disorders

The fifth edition of the Diagnostic and Statistical Manual of Mental Disorders (DSM-V) characterizes depressive disorders by the occurrence of “five or more symptoms during the same two-week period that are a change from previous functioning.” These symptoms include depressed mood, loss of interest or pleasure known as anhedonia, weight gain or loss, slowing down of thought, reduction of physical movement, fatigue, feelings of worthlessness, diminished ability to concentrate, and suicidal ideation. The DSM-V defines anxiety disorders as characterized by “excessive anxiety and worry (apprehensive expectation), occurring more days than not for at least 6 months” (Substance Abuse and Mental Health Services Administration, 2016). Anxiety disorders include generalized anxiety disorder, social anxiety disorder, panic disorders, and specific phobias.

The World Health Organization ranks depression as the leading cause of disability worldwide, quantified by the number of years lived with disability (YLDs) (World Health Organization, 2017). Depressive disorders contribute ∼4.4% to all YLDs. Anxiety disorders are ranked sixth, contributing to ∼3.4% of YLDs. Over 300 million people worldwide experience depression and >250 million people an anxiety disorder. Mood and anxiety disorders are often comorbid (Khan et al, 2005) as concurrent diagnoses occur in upward of 70% of patients (Essau, 2008). According to the Anxiety and Depression Association of America, anxiety disorders are the most common mental illness in the United States, affecting 40 million individuals annually. Limited progress has been made in the diagnosis and management of mood and anxiety disorders, largely because their etiologies remain fundamentally unknown.

### C Food and Drug Administration category classifications for medication use during pregnancy

Classifications of drugs for use during pregnancy emerged in 1979 after the thalidomide tragedy (Law et al, 2010). Thalidomide was introduced to the European market in 1957 as a safer alternative to barbiturates for treating insomnia (Kim and Scialli, 2011). Thalidomide was also marketed as a sedative for children and to pregnant women for vomiting, nausea, and morning sickness (Kim and Scialli, 2011). Thalidomide was widely used during the 1950s and 1960s in European countries but was never introduced to the American market due to the efforts of the FDA medical officer Frances Oldham Kelsey (Kim and Scialli, 2011). Concerned about a lack of safety data, Kelsey refused to authorize thalidomide for use in the United States. As Kelsey correctly suspected, thalidomide use during pregnancy was subsequently shown to cause birth defects, predominantly phocomelia, that is, severe malformations of the extremities, in thousands of exposed babies (Kim and Scialli, 2011).

In response to thalidomide, the FDA implemented labeling requirements for medications used during pregnancy in 1979. Drugs fall into 1 of the following 5 categories—A, B, C, D, or X. Drugs in categories A and B are associated with well-controlled studies in pregnant women or animal studies wherein minimal or no fetal risks have been detected (Law et al, 2010). Drugs in categories C and D have some documented fetal risk associated with their use, but their benefits may outweigh their risks. Drugs in category X should not be used during pregnancy, as possible benefits are greatly outweighed by the risks of use (Law et al, 2010). Approximately 60% of all drugs fall into category C, highlighting the lack of research surrounding the safety of drug use during pregnancy (Bourke et al, 2014; Pernia and DeMaagd, 2016). As discussed further, physiologic, pharmacokinetic, hormonal, and behavioral changes occur during pregnancy. As a result, studying the effects of medications specifically in pregnant subjects is crucial for determining safety.

## II Pregnancy-induced changes that affect drug action

Changes occur in all body systems during pregnancy, including in the cardiovascular, gastrointestinal, and neuroendocrine systems (Costantine, 2014; Soma-Pillay et al, 2016). These alterations are particularly relevant for drug metabolism. Moreover, the mammalian placenta produces new hormones and expresses its own receptors transporters, which change drug pharmacokinetics and internal homeostasis.

### A Pharmacokinetics

#### 1 Absorption

Absorption is the process of transporting a drug from its site of administration to the systemic circulation (Currie, 2018; Alagga and Gupta, 2022). Mechanisms involved in absorption include passive diffusion, carrier-mediated membrane transport (active transport and facilitated diffusion), and uptake by nonspecific drug transporters, for example, P-glycoprotein (Isoherranen and Thummel, 2013; Alagga and Gupta, 2022). Factors such as route of administration, gastric pH, and drug lipophilicity and molecular size affect bioavailability, that is, how much of a drug is available in the circulation after nonintravenous administration, for example, oral, intranasal, and transdermal (Feghali et al, 2015; Alagga and Gupta, 2022). Drugs taken orally undergo a first-pass effect, wherein they are metabolized by gastrointestinal organs, primarily the liver, reducing overall bioavailability (Feghali et al, 2015).

Pregnancy results in decreased gastrointestinal motility and increased gastric pH, which affect drug absorption after oral administration (Isoherranen and Thummel, 2013). Reduced gastrointestinal motility delays the absorption of drugs, while increased gastric pH deprotonates some drugs, which reduces absorption. Vomiting is a common symptom during pregnancy, particularly in the first trimester. Emesis may reduce drug concentrations, especially with the oral route of administration (Feghali et al, 2015).

#### 2 Distribution

Distribution is the process by which drugs move from the bloodstream to the tissues (Currie, 2018). Factors such as blood plasma protein binding and membrane permeability affect distribution. For instance, drugs that are tightly bound by plasma proteins have reduced tissue availability. The blood–brain and blood–placenta barriers actively reduce drug entry to the brain and fetus, respectively. The volume of distribution (Vd) is a theoretical volume used to indicate how extensively a drug will distribute in the body (Feghali et al, 2015). A high Vd indicates high distribution and low plasma protein binding.

During late pregnancy, hepatic production of glucose increases yet fasting blood glucose levels decrease (Lain and Catalano, 2007). Approximately one-third of the increased glucose is used by uterine, fetal, and placental tissues. Increases in adipose tissue result in average body weight gains of 3.5 kg (Lain and Catalano, 2007). Adipose tissue increases may result in higher volumes of distribution of lipophilic drugs, for example, SSRIs, thus leading to changes in half-life (Feghali et al, 2015). Maternal plasma volume increases throughout pregnancy as does cardiac output, while drug-plasma protein binding decreases (Notarianni, 1990; Jeong, 2010). Together, these changes result in a higher Vd for lipophilic drugs, yet reduced plasma concentrations of drugs (Deligiannidis et al, 2014). Blood flow to the uterus also increases 10-fold, and low molecular weight lipophilic drugs readily cross the fetal–placental barrier (Feghali et al, 2015). This leads to a build-up of lipophilic drug concentrations in the amniotic fluid, providing another source of fetal exposure to drugs, in addition to circulation via placental passage (Hostetter et al, 2000; Loughhead et al, 2006).

#### 3 Metabolism

Metabolism is the process by which drugs are modified by enzymatic processes, typically in the gastrointestinal tract or liver (Currie, 2018). Metabolism of certain medications is greatly affected by enzyme isoforms, particularly enzymes belonging to the cytochrome P (CYP) 450 family, during phase I metabolism (Deligiannidis et al, 2014). Phase I reactions are oxidation, reduction, or hydrolysis reactions, which make drugs more hydrophilic. In phase II, conjugation reactions occur, such as sulfation and glucuronidation, which increase the size and hydrophilicity of drugs to facilitate excretion (Isoherranen and Thummel, 2013).

Pharmacogenomics—the study of how genetic variations affect drug response—is useful in understanding maternal–fetal dynamics with respect to drug exposure during pregnancy (Blumenfeld et al, 2010). For example, key findings have identified ultrafast metabolizers versus intermediate or poor metabolizers of SSRIs and how these differences may be exaggerated during pregnancy (Ververs et al, 2009). Progesterone levels, which increase during pregnancy, induce greater CYP3A4 activity accelerating the metabolism of drugs metabolized by CYP34A, for example, fluoxetine, paroxetine, venlafaxine, and bupropion (Deligiannidis et al, 2014). Contrasting with the increased activity of CYP3A4, CYP2C19 activity is reduced by almost half during pregnancy, which is particularly important for the metabolism of citalopram and escitalopram (Deligiannidis et al, 2014). Reduced activity of CYP2C19 results in elevated drug-plasma concentrations.

#### 4 Excretion

Excretion eliminates drugs and their metabolites from the body, through not only urine or feces but also exhalation or sweat (Currie, 2018). Renal, cardiac, and hepatic actions affect overall clearance rates. Steady-state drug concentrations are determined by drug doses and clearance rates. Clearance from plasma circulation reduces overall drug concentrations and half-lives. During pregnancy, glomerular filtration rates increase, thereby increasing renal clearance. Increased renal clearance leads to decreased drug concentrations. In a study, increased renal clearance translated to increased depression scores, necessitating antidepressant dose adjustments (Sit et al, 2008). Pharmacokinetic changes during pregnancy are summarized in Table 1.

**Table 1:** Pharmacokinetic changes during pregnancy

Pharmacokinetic Property	Change During Pregnancy	Consequence
Absorption	• Increased gastric pH • Decreased gastrointestinal motility • Altered CYP450 activity	• Altered systemic absorption • Altered bioavailability
Distribution	• Increased plasma volume and cardiac output • Reduced drug-plasma protein binding, such as to albumin • Increased adipose tissue	• Reduced drug concentrations • Increased volume of distribution for lipophilic drugs
Metabolism	• Phase I and II enzymatic changes	• Altered drug metabolism, especially of drugs metabolized by CYP450 enzymes
Excretion	• Increased renal clearance	• Reduced steady-state drug concentrations • Increased elimination of drugs cleared by the kidneys

### B Neuroendocrine changes

Pharmacokinetics are also influenced by pregnancy-related hormonal changes (Jeong, 2010). Hypothalamus–pituitary–adrenal (HPA) hormones are important in this regard, as are hormones secreted by the placenta.

The HPA axis is an important modulator of stress responses (Zoubovsky et al, 2020). Activation of the HPA by stress increases the synthesis and release of hypothalamic and pituitary hormones, which amplify cortisol release from the adrenal glands. During pregnancy, corticotropin-releasing hormone (CRH), in the hypothalamus, increases from concentrations of <200 pg/mL to concentrations of >1000 pg/mL in the plasma (Sandman and Davis, 2012). The main function of CRH is to stimulate the synthesis of adrenocorticotropic hormone (ACTH) produced by the pituitary gland. The release of ACTH stimulates the synthesis of cortisol, glucocorticoids, mineralocorticoids, and androsterone.

While cortisol release by the adrenal glands normally negatively regulates hypothalamic and pituitary production of their respective hormones, during pregnancy, cortisol leads to the stimulation, synthesis, and release of cortisol, ACTH, and CRH in the fetus (Sandman and Davis, 2012). The placenta also expresses genes for CRH; however, placental CRH and hypothalamic CRH genes have nearly opposite responses because of differential regulation by transcription factors. The placenta can “sense” stress-related changes leading to adverse effects, for example, preterm birth or pre-eclampsia (Dimasuay et al, 2016). Furthermore, postpartum depression, which is associated with responses to stress, may affect childcare, leading to reduced maternal engagement with the baby, greater unpredictability in routines, and reduced breastfeeding duration (Becker et al, 2016).

Prospective cohort studies correlating maternal cortisol levels and offspring health are scare, yet provide key insights linking maternal cortisol levels with child behavioral outcomes. A study in 2009 found that maternal serum cortisol levels were negatively correlated with IQ scores in children aged 7 years (LeWinn et al, 2009). In a 2019 study conducted on 163 mother–child dyads recruited from Emory Women’s Mental Health Program, Swales et al (2018) found that elevated cortisol levels at 24 weeks of gestation predicted heightened emotional reactivity in children at an average age of 44 months. In a 2023 study published in *The Lancet*, Shriyan et al (2023) found that cortisol levels of >18 *μ*g/L were significantly associated with low infant birth weight and increased postpartum depressive symptoms in patients recruited from a public health facility in India.

Other hormones influence maternal and fetal physiology. For example, maternal estrogen, progesterone, and aldosterone levels increase during pregnancy. Progesterone downregulates maternal gastrointestinal motility, which delays the absorption of orally administered drugs (Jeong, 2010). Estrogen and progesterone regulate CYP enzyme expression, thereby influencing drug metabolism. Maternal growth hormone (GH) levels also decrease, but other GHs produced by the placenta increase, as discussed further (Jeong, 2010).

### C The placenta

During pregnancy, an entirely new organ is formed—the placenta. The placenta plays important roles in protecting the fetus from maternal immune responses, supplying nutrients, and facilitating gas exchange between fetal and maternal circulations (Griffiths and Campbell, 2015). The placenta prevents maternal immune system activation to paternal antigens expressed by fetal cells (Napso et al, 2018). For example, the placenta promotes several regulatory mechanisms, for example, altered antigen presentation and T cell differentiation, which create “distractions” for the maternal immune system, thereby enabling fetal viability (Than et al, 2019). Moreover, maternal IgG antibodies cross the placental barrier and provide passive immunity for the fetus (Wessel and Dolatshahi, 2024).

**Fig. 1:**
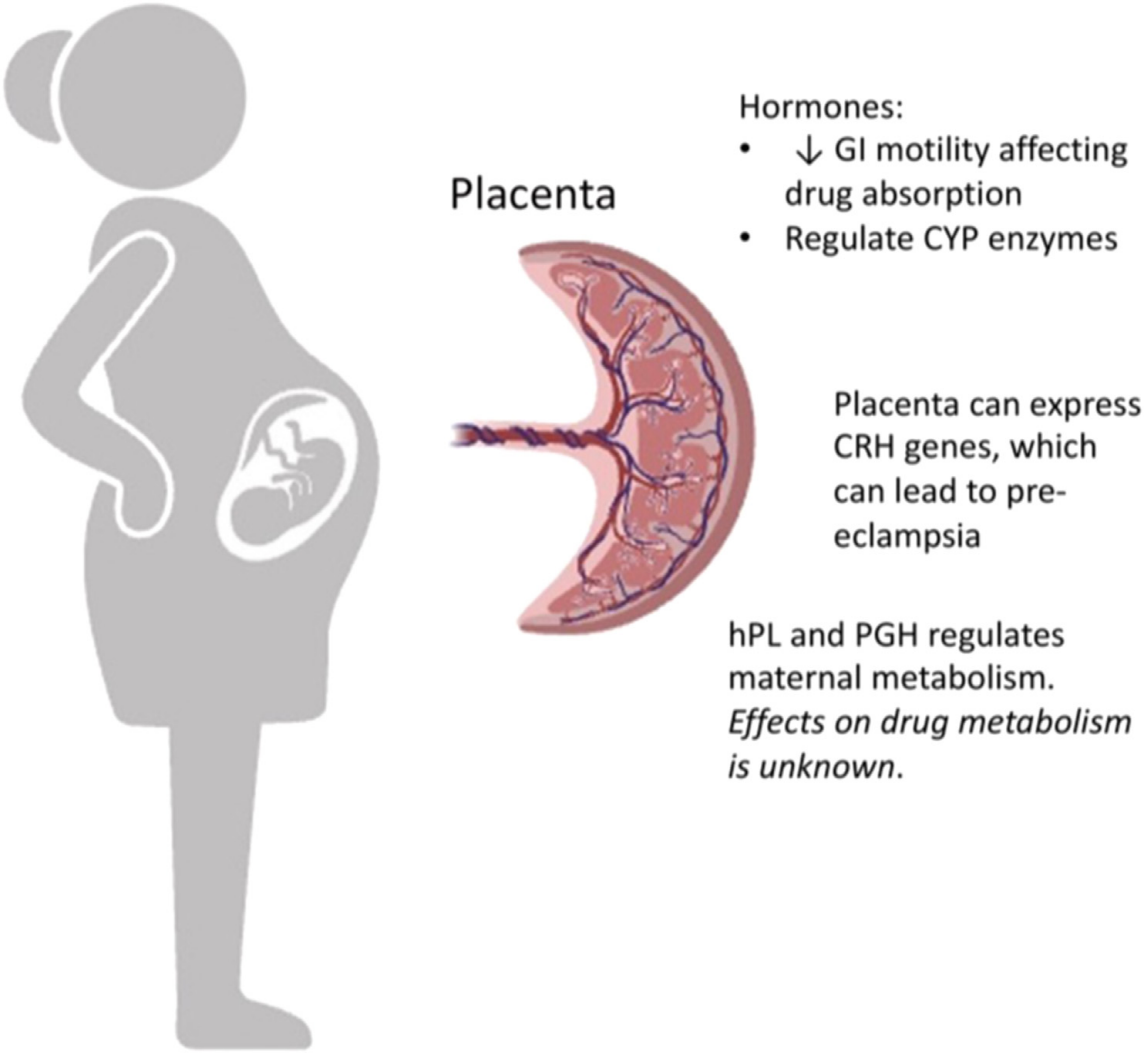
Placental impact on drug metabolism via hormones. Placental secretion of placental hormone (hPL) and placental growth hormone (PGH) regulates maternal metabolism, yet little is known about their effects on drug metabolism and other pharmacokinetic parameters. GI, gastrointestinal.

The placenta is a highly active endocrine organ (Napso et al, 2018). It produces and secretes human placental hormone and placental GH, which regulate maternal metabolism, for example, lipolysis promotion in adipose tissue. Levels of human placental hormone and placental GH increase 30-fold (∼1 g/d, the greatest of any known human hormone) and 100-fold (∼14 ng/mL after week 28), respectively, during pregnancy, yet little is known about their overarching effects, especially on drug metabolism and other pharmacokinetic parameters (Jeong, 2010; Velegrakis et al, 2017). Figure 1 provides a summary of placental–drug metabolism interactions.

The placenta is the sole link between mother and fetus. Drugs that cross the maternal blood–placental barrier will reach the fetus (Griffiths and Campbell, 2015). Three types of drug transfer occur (Pacifici and Nottoli, 1995). Type 1 drugs rapidly cross the placenta and significant concentrations are observed in maternal and fetal plasma. Type 2 drugs reach greater concentrations in the fetus than the mother, for example, ketamine (Ellingson et al, 1977). Type 3 drugs have incomplete transfer, resulting in higher concentrations in the maternal plasma than that in fetal plasma. Many factors affect drug transfer, including placental surface area and thickness, drug molecular weight and lipid solubility, and the pH of maternal and fetal blood (Griffiths and Campbell, 2015). Drug transfer occurs through placental passive or facilitated diffusion, active transport, or pinocytosis (Griffiths and Campbell, 2015). Antidepressants like SSRIs inhibit some placental–drug transporters, for example, P-glycoprotein, which bind numerous endogenous ligands, including cortisol and aldosterone. As such, inhibition of P-glycoprotein induces alterations in fetal exposure to maternal hormones (Feghali et al, 2015).

In sum, trimester-specific pharmacokinetic, hormonal, and anatomical changes occur during pregnancy. Factors such as the onset of maternal depression or anxiety, time frame of antidepressant administration, and drug dose will need rigorous investigation because they may explain discrepancies, that is, conflicting evidence between studies on physiologic, psychomotor, behavioral, and cognitive risks, associated with current knowledge about in utero antidepressant exposure on fetal and infant health. To the authors knowledge, no studies have rigorously compared the efficacy of antidepressant treatment before and during pregnancy to assess the effects of pregnancy-induced physiologic changes. Moreover, no studies have rigorously compared hormone levels before and after antidepressant exposure during pregnancy to monitor specific hormonal changes.

## III Origins of the monoamine hypothesis of depression: first-generation antidepressants

In the 1940s and 1950s, depressive disorders were treated by invasive brain procedures, leading to side effects and permanent disability. The history of treating psychiatric disorders is one of inhumane treatment, lack of informed consent, and serendipity (Staudt et al, 2019). While electroconvulsive therapy had been the primary treatment for depressive disorders, in the 1950s, lobotomies were proposed as a more effective alternative, particularly for patients with severe depression (Cheng et al, 1956). Pharmacotherapies took a center stage for treating mood and anxiety disorders after the downfall of Walter Freeman, a neuroscientist who developed and used transorbital lobotomies (Caruso and Sheehan, 2017). Transorbital lobotomies did not require surgery, so Freeman performed the procedure alone after his partner, neurosurgeon James W. Watts, refused to carry out lobotomies owing to their lack of safety. Freeman performed lobotomies on >4000 patients despite his lack of formal surgical training. Additionally, he carried out transorbital lobotomies on 2500 patients.

Antipsychotic medications were discovered in the 1930s, in parallel with the rise of ECT and lobotomies. Chlorpromazine, a first-generation antipsychotic, was approved for use in 1955 (López-Muñoz et al, 2005), turning the tide away from invasive procedures to drugs as therapeutics for mood and anxiety disorders (Caruso and Sheehan, 2017). In the 1950s, major breakthroughs for antidepressants occurred (Staudt et al, 2019). Physicians noted that tuberculosis medications, namely isoniazid and iproniazid, improved the mood of hospitalized patients with tuberculosis. Isoniazid and iproniazid were soon discovered to be MAOIs (Tretter, 2010). Monoamine oxidase is responsible for the metabolism of monoamine neurotransmitters, that is, dopamine, norepinephrine, and serotonin (Ramachandraih et al, 2011). The MAOIs prevent degradation of these transmitters, thereby increasing their concentrations in brain tissue (Andrews and Murphy, 1993) and prolonging their duration of action in the extracellular space. The MAOIs are still used to treat psychiatric disorders, such as anxiety and depression that is refractory to improvements SSRIs or SNRIs. Tricyclic antidepressants (TCAs) were subsequently discovered to inhibit the reuptake of monoamine neurotransmitters. Together, MAOIs and TCAs are referred to as first-generation antidepressants.

The fact that MAOIs and TCAs interacted with the dopamine and norepinephrine systems and improved mood and reduced anxiety led to the catecholamine hypothesis of depression proposed by Schildkraut, Bunney, and Davis in 1965 (Schildkraut, 1995; Pereira and Hiroaki-Sato, 2018). A role for serotonin came later when the TCA imipramine was discovered to inhibit serotonin reuptake, in addition to norepinephrine reuptake. Because some TCAs were shown to inhibit serotonin reuptake and MAOIs impacted serotonin catabolism, Coppen (1967) proposed that serotonin was important in the mood-improving properties of these drugs. The serotonin hypothesis of depression purports that decreased serotonin levels are a cause of depression. Based on the evolving understanding of the complex roles of serotonin in depression and the fact that MAOIs and TCAs had many adverse side effects, for example, seizures and cardiac dysfunction, pharmaceutical companies set out to discover drugs that selectively impacted the serotonin system.

While research during pregnancy is lacking, MAOIs and TCAs are, nonetheless, not recommended for use during pregnancy (Ram and Gandotra, 2015). Some drugs from each class fall under category C by the FDA. Of the TCAs, amitriptyline (Elavil), amoxapine (Asendin), clomipramine (Anafranil), and trimipramine (Surmontil) have the classification C (of the 9 FDA-approved TCAs for the treatment of depression), while the remaining TCAs are not reported owing to a lack of well-controlled studies in pregnant people (O’Connor et al, 2019). Of the MAOIs, isocarboxzid (Marplan) and selegiline (Ensam, a transdermal patch) have a category C classification (of the 4 FDA-approved MAOIs for the treatment of depression) (O’Connor et al, 2019). Because MAOIs and TCAs have significant off-target effects, they are mainly used for TRD in favor SSRIs and SNRIs, which have far fewer side effects (discussed further). The side effects of MAOIs and TCAs arise from their actions on adrenergic, cholinergic, and histaminergic systems (Chu and Wadhwa, 2024). The MAOIs pose a high risk of a hypertensive crisis, and both MAOIs and TCAs have many known contraindications with food and other drugs (Moraczewski et al, 2024; Sub Laban and Saadabadi, 2024).

## IV Second-generation antidepressants: serotonin reuptake inhibitors and serotonin norepinephrine reuptake inhibitors

In 1972, the pharmaceutical company Eli Lily reported on the properties of fluoxetine, which was designated the most powerful and SSRI at the time (Wong et al, 2005). In 1987, fluoxetine was approved by the FDA for clinical use as an antidepressant, being marketed under the brand name Prozac. Fluoxetine is a second-generation antidepressant, along with all other SSRIs, SNRIs, and bupropion. Like other SSRIs, fluoxetine has fewer side effects compared with MAOIs and TCAs. However, it was ineffective or only partly effective for many patients despite the rapid rise in prescriptions.

The serotonin hypothesis is, at best, an oversimplification (Altieri et al, 2011). In fact, a review of the clinical literature finds little evidence to support the idea that reduced serotonin is associated with depression (Moncrieff et al, 2023). Nonetheless, the most prescribed medications used to treat depression and anxiety remain the SSRIs. All SSRIs are more effective than placebos, and 40%–60% of patients experience mood improvement upon sustained SSRI administration (Cipriani et al, 2018). Nonetheless, while depression is likely not caused by low brain (extracellular) serotonin, rodent models point to the serotonin transporter (SERT), which is the primary site for SSRI action, as a key modulator of serotonin transmission and anxiety-related behavior (Adamec et al, 2006; Wellman et al, 2007; Altieri et al, 2015; McHugh et al, 2015).

### A Statistics on use

In the United States, 13% of adults aged 18 years and older use antidepressants, mainly SSRIs. Of those who are pregnant, 1%–5% take SSRIs (Molenaar et al, 2020a). Effects of exposure to SSRIs are different depending on trimester, duration and consistency of use, and metabolic profiles, as discussed further. The SNRIs are another treatment option. In addition to their use in treating mood and anxiety disorders, SNRIs are also prescribed for chronic pain (Arnold et al, 2005, 2007; Clauw et al, 2008). Specific use statistics are difficult to obtain because SNRIs are generally lumped together with SSRIs and categorized under the umbrella term of antidepressant. Nonetheless, trends suggest that prescriptions of SNRIs, along with atypical antidepressants, are on the rise (Luo et al, 2020). The SNRIs are prescribed during pregnancy, although at lower rates than SSRIs, and, like SSRIs, are designated category C by the FDA (Pernia and DeMaagd, 2016).

### B Pharmacokinetics

The SSRIs, administered orally in pill or liquid capsule forms, include fluoxetine, citalopram (Celexa), escitalopram (Lexapro), sertraline (Zoloft), paroxetine (Paxil, Pexeva), and fluvoxamine (Luvox). In 2011 and 2013, vilazodone (Viibryd) and vortioxetine (Trentellix), respectively, were approved by the FDA for major depressive disorder, although they are the least prescribed SSRIs because of their shorter time in the market. The pharmacokinetic properties of the SSRIs are summarized in Table 2.

**Table 2:** Pharmacokinetic properties of SSRIs Bioavailability is the amount of unmetabolized drug that enters systemic circulation compared with that of intravenous administration. Volume of distribution is a measure of the ability of drug to redistribute from the plasma to other organs and tissues. Half-life is the amount of time required for the plasma drug concentration to be reduced by half. Steady-state is the half-life of the drug multiplied by 4.5 and reflects the time to a steady plasma drug concentration.

Medication	Bioavailability (F) %	Volume of Distribution (V_d_) L/kg	Half-life (t_1/2_) h	Enzymes in Metabolism	Time to Reach Steady-state
Fluoxetine	<90	20–45	1–4 d	CYP2D6	>3 wk
Fluvoxamine	∼50	∼5	8–28	CYP2D6	10 d
Citalopram	∼80	14–16	∼36	CYP2C19	6–10 d
Escitalopram	∼80	12–26	27–32	CYP3A4	7–10 d
Sertraline	∼44	20	22–37	CYP3A4	5–7 d
Paroxetine	<50	3–12	16–19	CYP2D6	7–14 d
Vilazodone	∼72	8	25	CYP3A4	∼5 d
Vortioxetine	∼75	37	66	CYP2D6	2 wk

All SSRIs undergo first-pass metabolism in the liver (van Harten, 1993). The SSRIs are metabolized by the CYP enzymes, primarily CYP2D6 and CYP3A4. Fluoxetine has the highest Vd and longest half-life compared with other SSRIs and has an active metabolite, norfluoxetine. Both fluoxetine and norfluoxetine have high affinity for SERT, as well as 5HT_2A_ and D2 receptors. Citalopram and escitalopram have the highest selectivity for SERT versus other monoamine reuptake transporters compared with other SSRIs. Almost all pharmacologic effects of citalopram are attributed to its (*S*)-enantiomer, escitalopram (Rao, 2007), which is sold under the brand name Lexapro (Hiemke and Härtter, 2000). Sertraline is unique among SSRIs because it also inhibits dopamine transporters. Some studies have investigated sertraline as a treatment for stimulant use disorders, as sertraline delays relapse rates compared with placebo (Oliveto et al, 2012; Chan et al, 2018). Vilazodone and vortioxetine, while potent SERT inhibitors, also show partial agonist activity at serotonin receptors (Cruz, 2012; D’Agostino et al, 2015).

The pharmacokinetic properties of SSRIs change during pregnancy (Anderson, 2005). For example, drug metabolism by some CYP isoenzymes increases (Betcher and Wisner, 2020). For paroxetine, which is exclusively metabolized by CYP2D6, women who are extensive or ultrarapid metabolizers showed decreased serum levels of paroxetine and significantly increased depression symptoms (Ververs et al, 2009). Intermediate or poor metabolizers showed increased paroxetine serum levels during pregnancy with no change in depressive symptoms. An ongoing study by the National Institute of Child Health and Development is examining how antidepressant concentrations change with respect to physiologic changes during pregnancy and during the postpartum period (Clinical Trail NCT02519790). Wisner et al (2024) recently published a communications article urging the recognition of the role of mental illness in maternal mortality.

Like SSRIs, SNRIs are administered orally via pills or liquid capsules. The family of SNRIs consists of duloxetine (Cymbalta), desvenlafaxine (Pristiq), levomilnacipran (Fetzima), milnacipran (Savella), and venlafaxine (Effexor, discontinued). The SNRIs were approved for use by the FDA in the early 2000s, except venlafaxine, which was approved in 1993 (immediate-release [IR] formulation) and 1997 (extended-release) and levomilnacipran in 2013. Milnacipran is the only SNRI not used for the treatment of major depression or anxiety disorders; rather, it is almost exclusively prescribed for the treatment of fibromyalgia (Clauw et al, 2008). The half-life of the SNRIs is ∼10 hours (Sansone and Sansone, 2014). All SNRIs except milnacipran require metabolism by liver CYP enzymes. Milnacipran bypasses CYP metabolism and undergoes phase II conjugation. The bioavailability and Vd of SNRIs are similar to the SSRIs (Sansone and Sansone, 2014). Pharmacokinetic properties of SNRIs are summarized in Table 3.

**Table 3:** Pharmacokinetic properties of SNRIs.

Medication	Half-life (t_1/2_) h	Enzymes in metabolism	Preferential affinity for NET or SERT
Venlafaxine	11–14	CYP2D6	30× higher affinity for SERT vs NET
Desvenlafaxine	11	CYP3A4	10× higher affinity for SERT vs NET
Levomilnacipran	12	CYP3A4	3× higher affinity for NET vs SERT
Milnacipran	8–10	Phase II conjugation	No preference for NET vs SERT
Duloxetine	12	CYP2D6	10× higher affinity for SERT vs NET

### C Pharmacodynamics

The SSRIs act on SERTs, blocking the reuptake of serotonin from the extracellular space into presynaptic neurons and other cell types, for example, enterochromaffin cells in the gut. While some SSRIs, such as paroxetine and sertraline, have affinity for other monoamine transporters, the SSRIs are relatively selective for SERT. The SNRIs target both serotonin and norepinephrine transporters (NETs) with high affinity, thereby blocking the reuptake of both monoamines (Sansone and Sansone, 2014). The SNRIs also impact extracellular dopamine levels indirectly. In the prefrontal cortex, dopamine is predominately taken up by NET instead of the dopamine transporter, which is expressed at low levels. Thus, SNRIs work as triple uptake inhibitors, to some extent, via inhibition of dopamine reuptake by NET in the prefrontal cortex (Morón et al, 2002).

Some SNRIs are used to treat chronic pain conditions, such as chronic musculoskeletal pain, diabetic peripheral neuropathic pain, and fibromyalgia (Marks et al, 2009). Both duloxetine and milnacipran are prescribed for fibromyalgia. Effectiveness in treating chronic pain implicates norepinephrine and serotonin transmission in chronic pain (Stahl, 2009). The raphe nuclei, where the serotonergic cell bodies are located, and the locus coeruleus, the site of most norepinephrine cell bodies, send their projections to the dorsal horn of the spinal cord via the dorsolateral funiculus (Fields et al, 1991). Serotonin and norepinephrine descending fibers suppress pain transmission by hyperpolarization of afferent sensory neurons, preventing the relay of nociception to the thalamus and, eventually, cortical regions (Marks et al, 2009). Increased extracellular norepinephrine and serotonin increase inhibition of ascending pathways. Serotonin-mediated and norepinephrine-mediated inhibition of these pathways may lead to their therapeutic effects in chronic pain conditions.

Microdialysis and voltammetry studies have confirmed that serotonin reuptake inhibitors produce elevated extracellular serotonin concentrations in a matter of minutes (Movassaghi et al, 2021; Dagher et al, 2022). Yet, for most patients, SSRIs take 1–6 weeks to improve symptoms, if improvements occur at all (Taylor et al, 2006). Thus, the mechanisms by which SSRIs improve mood and anxiety involve effects beyond their immediate action at SERT. Potential therapeutic mechanisms include prolonged increases in extracellular serotonin, desensitization/downregulation of serotonin1A autoreceptors, and increased brain-derived neurotrophic factor (BDNF), synaptogenesis, and neurogenesis, which are discussed subsequently (Taylor et al, 2005; Pittenger and Duman, 2008; Castrén and Hen, 2013; Liu et al, 2017).

### D Fetal exposure and risk associated with serotonin reuptake inhibitors use

The SSRIs and SNRIs cross the placental–fetal barrier, although not in similar ways (Hendrick et al, 2003). A 2003 report of umbilical cord SSRI concentrations from 38 women found that maternal doses of sertraline and fluoxetine were significantly correlated with umbilical cord serum drug concentrations (Hendrick et al, 2003). This correlation, however, was not observed for citalopram. The SSRIs and SNRIs have also been detected in the amniotic fluid, which the fetus swallows, providing another source of fetal exposure (Hostetter et al, 2000; Loughhead et al, 2006). Finally, babies are exposed to SSRIs via breast milk, although concentrations are low (often undetectable) and may not be of clinical relevance (Suri et al, 2002; Weissman et al, 2004; Payne, 2019). Three potential fetal risks that have been identified are discussed further. These are persistent pulmonary hypertension (PPHN), neonatal adaptation syndrome (NAS), and congenital malformations.

In 2006, the FDA issued a health advisory warning against the use of SSRIs during pregnancy owing to an increased risk of PPHN, a dangerous condition where fetal circulation does not properly transition after birth. When adjusting for confounding factors, such as the severity of maternal mood or anxiety disorder, the risk of PPHN was subsequently determined to be minimal to none, leading the FDA to rescind this warning in 2011 (Occhiogrosso et al, 2012). The risk of teratogenesis is low, and there are no specific patterns of major malformations (Bourke et al, 2014) Paroxetine is the only SSRI in category D (vs C) owing to reports of an increased occurrence of cardiac malformations in infants exposed to paroxetine during the first trimester (O’Connor et al, 2016). Cardiac malformations induced by paroxetine appear to be dose and trimester specific (Marks et al, 2008).

A 2020 retrospective cohort study examined SSRI exposure in the context of prenatal and placental outcomes. This study found decreased birth weights, increased adverse neonatal outcomes, for example, hypoglycemia and seizures, and reduced placental weights in newborns exposed to maternal SSRIs during pregnancy (Levy et al, 2020). However, like most studies on SSRI exposure during pregnancy, there was limited information about the type of SSRI, duration, or dose (Levy et al, 2020). In most studies, increased spontaneous abortion rates were reported in conjunction with maternal antidepressant treatment, whether SSRIs, SNRIs, or atypical antidepressants were at issue (Gentile, 2005a). As discussed further, these studies were confounded by the occurrence of maternal psychiatric disorders, which by themselves producing adverse neonatal outcomes (Coussons-Read, 2013).

Infants exposed to SSRIs in utero during late pregnancy experience withdrawal at birth (Gentile, 2005a). Withdrawal leads to NAS (Perinatal Services, 2013). Up to 70% of infants develop a spectrum of NAS symptoms, including jitteriness, motor hyperactivity, irritability, and a weak cry (Galbally et al, 2017). Some hypothesize that NAS is the result of withdrawal from maternal medication. Others hypothesize that NAS results from overstimulation of the serotonergic system, leading to toxicity from increased serotonin concentrations. Regardless of the cause of NAS, its symptoms are self-limiting, normally dissipating in as little as hours. The NAS syndrome does not seem to have prolonged effects on infant health outcomes (Galbally et al, 2017).

Risks associated with SNRI use are similar to those of SSRIs. All SSRIs and SNRIs can cause serotonin syndrome and are contraindicated for use with MAOIs (Sub Laban and Saadabadi, 2022). Serotonin syndrome is a potentially fatal side effect of increased serotonin concentrations, although it rarely occurs with SSRI use during pregnancy (Boyer and Shannon, 2005; Fox et al, 2009).

### E Longitudinal cohort studies

Most studies examining the effects of antidepressants during pregnancy are observational and thus, only useful for descriptive information. The most common observational studies on the association between antidepressants and adverse fetal outcomes are either case–control or cohort studies (McDonagh et al, 2014; Munnangi and Boktor, 2022). While observational studies provide important insights about how exposures, for example, to SSRIs, affect offspring outcomes such as birth defects, these types of studies are associated with confounding factors that prevent clear study conclusions (Griesdale and Jones, 2018). Broadly, confounding occurs when the presence of a variable other than the exposure of interest influences the estimated effect of exposure on a given outcome (Griesdale and Jones, 2018). A confounding variable is one that is associated with both the exposure and the outcome (Griesdale and Jones, 2018).

Lack of strong evidence surrounding antidepressant safety risks, particularly SSRIs, advises against discontinuing antidepressant use during pregnancy (Jimenez-Solem, 2014; Molenaar et al, 2018; Kolding et al, 2021; Trinh et al, 2023). Many studies have found no adverse effects on infant neurobehavioral outcomes upon perinatal exposure to antidepressants (Gentile, 2005b; Misri et al, 2006; Wisner et al, 2009; Smith et al, 2013; Eriksen et al, 2015; Hutchison et al, 2019b). Recently, a 2022 cohort study investigated the association between antidepressant use during pregnancy and risk of neurodevelopmental disorders in children; the authors found no association with autism spectrum disorders, learning disabilities, and behavior disorders, among many other outcomes, in a cohort of >140,000 children exposed to antidepressants during gestation (Suarez et al, 2022).

Other studies report adverse effects, particularly when looking at outcomes involving motor inhibitory control and birth weight (Mulder et al, 2011; Smith et al, 2013; Santucci et al, 2014; Molenaar et al, 2020b; van der Veere et al, 2020). In a study by Santucci et al (2014), infant psychomotor development was significantly different in infants exposed to perinatal antidepressants at 26 and 52 weeks after birth. Motor differences were no longer observed at 78 weeks, suggesting transient self-correcting changes (Santucci et al, 2014). In a 2020 study, motor function in children exposed to antidepressants was no longer significantly different from children in the unexposed group when adjusting for the severity of maternal anxiety (van der Veere et al, 2020). Risk for preterm birth is increased in individuals taking SNRIs compared with those taking SSRIs (Lennestål and Källén, 2007). However, preterm birth has also repeatedly been associated with maternal depression (Bourke et al, 2014). In fact, a cohort study conducted by Amit et al (2024) used data from >200,000 electronic health records in the United Kingdom and found that while a history of maternal depression was associated with preterm birth, antidepressant exposure during gestation was not.

Another 2020 study found that exposure to in utero antidepressants increased the odds of poor developmental health, measured by the Early Development Instrument survey, in kindergarteners (Singal et al, 2020). Developmental vulnerability was seen in ∼20% of exposed children versus 16% of children born to depressed mothers who did not take SSRIs or SNRIs. The limitations of this study include a lack of control for factors such as disease severity, specific antidepressant medications, and time of gestational exposure (Singal et al, 2020). Notably, many of the complications observed in children of depressed mothers are often reported with antidepressant exposure, pointing to the confounding nature of underlying maternal pathology (confounding by indication) (Burt and Quezada, 2009). For example, a population-based cohort study of children born from 2006 to 2007 in Sweden found that intellectual disability reported in infants exposed to antidepressants in utero was likely attributed to underlying maternal depression (Viktorin et al, 2017).

Maternal depression during pregnancy is associated with several complications, including preeclampsia, low birth weight, and premature birth (Becker et al, 2016). A recent longitudinal study found that exposure to maternal depression adversely affects the developing executive function of children at 3 and 6 years of age (Hutchison et al, 2019a). Moreover, antenatal depression and anxiety directly impact postpartum parenting stress, which can negatively impact parent–child relationships (Misri et al, 2010). In a systematic review, Rommel et al (2020) found that underlying maternal disorders drove reported associations of neurodevelopmental, physical, and psychiatric fetal and infant outcomes. The effects of maternal depression or anxiety are exacerbated in non-White families, particularly those with low-income status (Nillni et al, 2018). Overall, when examining the risk of antidepressants in offspring, conflating risks posed by maternal mood and anxiety disorders often falsely attributes risks to antidepressants.

Another factor that significantly alters epidemiologic findings is control group selection (Andrade, 2017). Control groups are often comprised healthy women with no psychiatric diagnoses. Some control groups included women receiving psychotherapy instead of pharmacotherapy, women in remission from depression and anxiety disorders at the time of their pregnancy, or matched siblings with no psychiatric disorders to account for genetic and environmental variability (Andrade, 2017). Another control group is women with depression who pause their antidepressant before getting pregnant, compared with those who continue antidepressant treatment during pregnancy. Studies that use this control group have also indicated that antidepressant use during pregnancy does not pose increased fetal, neonatal, teratogenic, or developmental risks, especially when considering confounding by inidication (Lee and Chang, 2022; Besag and Vasey, 2023; Smith et al, 2024). In a study, McDonagh et al (2014) concluded that more specific treatment comparisons, for example, specific SSRIs, severity of depression, and factors such as drug timing and dose and outcomes assessments by blinded evaluators, are needed to draw concrete conclusions. These authors advocate for including pregnant women in randomized controlled trials (RCTs).

While longitudinal studies provide meaningful information, risk due to antidepressant exposure during pregnancy would be better assessed using RCTs. Nonetheless, RCTs pose ethical concerns when conducted during pregnancy. Neonatal safety is a major issue that often pits maternal health against fetal exposure. Only in 1993 did the FDA lift the ban on pregnant women participating in RCTs. A lack of pregnant women in RCTs has resulted in a general lack of established precedent for medication safety during pregnancy (Unger et al, 2011).

Prospective cohort studies can address limitations of longitudinal studies by incorporating better control groups, specific inclusion and exclusion criteria, and by controlling for relevant variables that influence infant outcomes, for example, socioeconomic status, race and ethnicity, and severity of maternal illness. Yet, because participants cannot be truly randomized and treatment length, medication type, and/or dose cannot be fully controlled, preclinical (animal) studies are warranted. To understand the causal effects of exposure to antidepressants, at least those common to mammals, studies using animal models are of importance.

### F Animal models for antidepressant use during pregnancy

Animal models have several advantages for investigating the biological and behavioral effects of maternal SSRIs on offspring. Simply, they enable specific drug and temporal manipulations without the difficulties of determining precisely how SSRIs were used by individual women. Drug type, dose, and timing are controlled by the investigators in preclinical studies. Moreover, genetic and environmental variability are more highly controlled (although not nonexistent) in animal studies (Butler-Struben et al, 2022). Thus, while not perfect models because of the developmental mismatches between rodents and humans, the use of animal models benefits from the ability to identify drug-outcome relationships in ways that longitudinal human studies cannot (Steimer, 2011; Semple et al, 2013).

Stress is one of the most commonly identified risk factors that predisposes women to develop and maintain mood and anxiety disorders (Coussons-Read, 2013). In animals, stress induces anxiety-like and depressive-like symptoms during or after pregnancy (Krishnan and Nestler, 2011). Paradigms that produce stress in laboratory animals include chronic unpredictable stress and social defeat stress (Krishnan and Nestler, 2011). The former uses ethologically relevant stressors that include predator odor, overnight light exposure (circadian rhythm disruption), and wet bedding or cage-tilt (nest insecurity) to elicit transient and unpredictable stress. When administered repeatedly, these stressors produce chronic stress (Willner, 2017; Sequeira-Cordero et al, 2019). In addition to chronic unpredictable stress and social defeat stress, maternal separation is used to assess the effects of poor caregiving during the postnatal period on preweaning pups (Roque et al, 2014).

Changes in behavior in mothers and offspring are assessed through an array of behavior tests to assess anxiety-like and depressive-like behaviors (Lezak et al, 2017). Tests such as the elevated plus maze, open field, and novelty suppressed feeding (NSF) tests are used to quantify anxiety-related behavior (Griebel and Holmes, 2013). The elevated plus maze, open field, and NSF tests place animals in an approach-avoidance conflict, for example, a brightly lit arena when an animal has been food deprived for 12–24 hours in the NSF test (Bach, 2022). The forced swim test (FST), tail suspension test (TST), and sucrose preference test are used to assess depressive-like behaviors, although these tests are generally less robust in their translational value (van der Staay et al, 2009; Ledford, 2014).

In the FST and TST, animals experience highly stressful situations for short periods, that is, mice or rats are briefly forced to swim in a cylinder of water and/or are suspended by their tails (Belovicova et al, 2017). While the FST, TST, and sucrose preference test, and particularly the FST, have been used extensively to predict antidepressant efficacy, the interpretation of their behavioral outputs remains contested (Ledford, 2014; Anyan and Amir, 2018). Still, when taken together, these tests provide information about behavioral changes between animal control and treatment groups even if the behavioral changes are open to interpretation.

## V Atypical antidepressants: bupropion and ketamine

The serotonin and norepinephrine pathways are connected to and work in concert with other transmitter systems. In mood disorders, reward and learning are impaired, and many patients experience depression characterized by the common symptom of anhedonia (Gorwood, 2008; Ng et al, 2019). Anhedonia refers to an inability to experience pleasure (Gorwood, 2008). From neuroimaging studies, the roles of the neurotransmistters dopamine and glutamate have emerged, particularly in the context of dysfunctional reward and deficits in learning (Gorwood, 2008).

Dopamine plays a role in neuropsychiatric pathologies where the reward system is impaired (Diehl and Gershon, 1992). Glutamate, which is the most abundant neurotransmitter, is important for synaptogenesis—the birth of new synaptic connections—and neuroplasticity—the overall flexibility or plasticity of neural connections (Rowley et al, 2012). Synaptogenesis and neuroplasticity are key factors in the therapeutic effects of antidepressants, as well as underlying healthy cognitive processes (Wilkinson and Sanacora, 2019). Thus, new avenues of research in the treatment of depression and anxiety are targeting other neurotransmitter systems, including the glutamatergic, dopaminergic, and cholinergic systems (Slemmer et al, 2000; Sanacora et al, 2008; Li et al, 2016).

### A The dopaminergic system and its targets

Dopamine is a monoamine neurotransmitter, like serotonin and norepinephrine. Dopamine transmission is implicated in mood and anxiety states. Evidence arises from studies on MAOIs, TCAs, mild stimulants, for example, Wellbutrin, Adderall, Ritalin, and drugs of abuse that lead to improved mood, energy, focus, and reduced negative states such as methamphetamine and cocaine, which primarily target dopamine transporter (Farzam et al, 2022). Dopamine cell bodies are located in the substantia nigra and ventral tegmental area, 2 neighboring brain nuclei that have different but overlapping projection profiles (Poulin et al, 2018). Dopaminergic projections target a number of brain regions, including the striatum, amygdala, prefrontal cortex, and hippocampus, and modulate brain processes, including reinforcement learning, reward, and mood (Poulin et al, 2018).

#### 1 Bupropion history and statistics on use

Bupropion (Wellbutrin) use has steadily increased over the last decade. Initially synthesized as an antidepressant, bupropion was also found to aid in smoking cessation and became FDA approved as a therapy for nicotine use disorder. While bupropion initially gained FDA approval in 1985, it was removed from the market owing to fears of increased seizure risks. With more careful dosing guidelines, bupropion was reintroduced, now being used by millions, primarily as an antidepressant (Stahl et al, 2004).

#### 2 Pharmacokinetics

Bupropion is extensively metabolized by liver CYP2B6 to its active metabolite hydroxybupropion (Foley et al, 2006; Deligiannidis et al, 2014). However, bupropion and hydroxybupropion inhibit CYP2D6, resulting in potential drug interactions. To a lesser extent, bupropion is metabolized by CYP2B6 enzymes located in the brain. The distribution of brain CYP2B6 is heterogeneous, leading to brain region-specific effects. For example, CYP2B6 is highly expressed in astrocytes in layer I of the frontal cortex and at the blood–brain interface, suggesting an important role for this enzyme in brain drug penetration and action (Ferguson and Tyndale, 2011).

Bupropion has a high lipid solubility and a low molecular weight leading to almost 100% absorption when taken orally (Foley et al, 2006). Nonetheless, bioavailability is only 5-20%. Low bioavailability has little impact on effectiveness because the active metabolite, hydroxybupropion has equal antidepressant effects to that of bupropion. The half-lives of bupropion and hydroxybupropion are ∼18 and ∼20 hours, respectively. Steady-state concentrations are achieved within 5–7 days of continuous dosing (Foley et al, 2006).

Bupropion is available as IR, sustained-release, and extended-release formulations (Foley et al, 2006). Details of bupropion formulations are summarized in Table 4. Differences in individual responses to bupropion, typically the IR formulation, influence which formulation is prescribed, as the different bupropion formulations have differing onsets of action and half-lives and, thus, durations (Jefferson et al, 2005).

**Table 4:** Bupropion formualtions and their properities

Bupropion Formulation	Dose mg	Frequency of Intake	Maximum Recommended Dose mg
Immediate-release	75 and 100	2× daily	450
Sustained-release	100, 150, and 200	1–2× daily	400
Extended-release	150 and 300	1× daily	450

#### 3 Pharmacodynamics

Bupropion acts as a reuptake inhibitor at dopamine and norepinephrine transporter (Stahl et al, 2004). Additionally, bupropion is a partial antagonist at nicotinic acetylcholine receptors, specifically *α*3*β*4 subunit–containing receptors (Slemmer et al, 2000). Bupropion is mechanistically distinct from SSRIs and SNRIs and, importantly, does not have direct effects on the serotonin system. A 2006 study compared the efficacy of sertraline (SSRI), venlafaxine (SNRI), and bupropion in patients who were treatment resistant to citalopram (SSRI) (Rush et al, 2006). The study concluded that there were no differences in the rates of remission between these 3 groups (Rush et al, 2006). Importantly, this study suggested that intolerance or lack of efficacy of 1 SSRI does not imply intolerance to or lack of efficacy of all SSRIs and that within-class (eg, SSRIs) and out-of-class (eg, SNRIs and bupropion), medication switches are reasonable choices.

Bupropion is commonly prescribed in addition to an SSRI, which seems to reduce sexual dysfunction associated with SSRI use and helps to improve remission rates (Clayton et al, 2004; Zisook et al, 2006). Discontinuation of bupropion generally stems from stimulatory effects, although its discontinuation rate is no different from other second-generation antidepressants, for example, SSRIs (Foley et al, 2006). A clinical study examined the use of sustained-release formulation of bupropion for the treatment of postpartum depression and found bupropion to be well-tolerated (no patients discontinued treatment) and more than half of the patients had improved mood scores (Nonacs et al, 2005). This study only had a small sample size (*N* = 8), limiting its findings (Nonacs et al, 2005). Currently, only allopregnanolone, a neurosteroid, is specifically (FDA, 2019b) approved for postpartum depression (Walton and Maguire, 2019). Moreover, to the authors knowledge, no study has compared the rates of postpartum depression given antidepressant treatment during pregnancy.

#### 4 Fetal exposure and risk associated with perinatal bupropion exposure

Bupropion and its metabolite hydroxybupropion cross the blood–placenta barrier and are retained in placental tissue (Earhart et al, 2010). Exposure to bupropion did not affect placental viability. Infant exposure via breastmilk is minimal for bupropion, only amounting to ∼2% of the maternal dose (Gentile, 2005a). Bupropion is prescribed during pregnancy and is a category C medication under the FDA classification system (O’Connor et al, 2016). This medication is indicated for antidepressant treatment and to assist with smoking cessation (Earhart et al, 2010).

A prospective cohort study compared pregnant women exposed to bupropion versus other antidepressants during the first trimester of pregnancy (Chun-Fai-Chan et al, 2005). Bupropion was not associated with increases in congenital malformations, gestational age at birth, or birth weight compared with other antidepressants. A higher rate of spontaneous abortions in the bupropion-exposed group compared with those not exposed to antidepressants was observed. However, like other studies, a limitation is separating whether increases in spontaneous abortions are a result of antidepressant use or underlying affective disorders (Chun-Fai-Chan et al, 2005). Overall, bupropion does not seem to produce teratogenic effects, although more research is needed (Gentile, 2005a).

### B Glutamatergic system and its targets

Glutamate is the most abundant neurotransmitter in the central nervous system. Glutamate transmission is important in synaptogenesis, functional connectivity between brain regions, and homeostasis (Sanacora et al, 2008). Key glutamatergic receptors include the NMDA and *α*-amino-3-hydroxy-5-methyl-4-isoxazolepropionic acid (AMPA) receptors, which are inotropic ligand-gated receptors, and metabotropic receptors—for example, mGLUR5—which are emerging as new therapeutic targets (Terbeck et al, 2015).

Glutamate is a nonessential amino acid and is required for the synthesis of the oppositional inhibitory neurotransmitter GABA. Termination of glutamate transmission requires glial reuptake, as opposed to reuptake via presynaptic neurons. These properties distinguish the glutamatergic system from the previously discussed monoamine systems (Sanacora et al, 2008; Rowley et al, 2012).

#### 1 Ketamine history and statistics on use

Atypical antidepressants have emerged most recently. In 2019, ketamine was FDA approved for TRD. The use of ketamine implicates neurotransmitter systems, in addition to the monoamines, in the treatment of depression and anxiety (FDA, 2019a). Ketamine was synthesized in 1962 by Calvin Stevens and gained FDA approval for human use as an anesthetic in 1970 (Jansen, 2000). Soon after, ketamine appeared on the illicit drug market and became widely abused. By the mid-1980s, ketamine became linked to “dance culture” and was used in a variety of social settings (Jansen and Darracot-Cankovic, 2001). In 2013 and 2016, esketamine, the (*S*)-enantiomer of ketamine, received the status of Breakthrough Therapy Designation for TRD and major depressive disorder. In 2018, a number of studies were published showcasing the ability of ketamine to improve mood rapidly in patients with TRD and to reduce rates of suicidality significantly (Canuso et al, 2018; Daly et al, 2018; Wilkinson et al, 2018). These findings resulted in a 2019 decision by the FDA to approve ketamine for TRD (FDA, 2019a).

#### 2 Pharmacokinetics

The effects of ketamine are almost instantaneous. With intravenous injection, the onset of action is ∼30 seconds. With intramuscular or intranasal administration, onset of action is still well <10 minutes. Ketamine is metabolized into its active and major metabolite norketamine in the liver by the enzyme CYP2B6 (Peltoniemi et al, 2016). Approximately 80% of ketamine is demethylated to norketamine, and norketamine is measured in blood plasma within minutes of intravenous ketamine administration (Peltoniemi et al, 2016).

At subanesthetic doses, ketamine produces rapid antidepressant effects, within 4 hours of administration (Berman et al, 2000). Doses of ketamine that produced antidepressant effects included 0.2 mg/kg intravenously, 0.25–0.5 mg/kg intramuscularly, and 50 mg intranasally (Abdallah et al, 2015). While single doses of ketamine via intravenous infusion produce rapid antidepressant effects, fear that these effects would not last prompted research into multiple infusions over longer timeframes (Shiroma et al, 2014). Now, the typical procedure is to receive 6 ketamine infusions over a span of several weeks (Blier et al, 2012). In addition to the intravenous route of administration, esketamine (Spravato) has been developed by Janssen Pharmaceuticals as an intranasal formulation (Canuso et al, 2018; Daly et al, 2018). A phase 4 clinical trial examined optimal intranasal dose for sustained antidepressant effects, although the findings are not yet published (clinical trial NCT04599855).

#### 3 Pharmacodynamics

Ketamine is a noncompetitive antagonist at NMDA receptors (Seeburg et al, 1995; Peltoniemi et al, 2016). The NMDA receptors are coincidence detectors, requiring intracellular depolarization to remove the magnesium ions that block the receptor channel pore and prevent ligand binding (Seeburg et al, 1995). Ketamine blocks the channel, thereby inhibiting receptor activation even in the presence of both events. The NMDA receptors are on GABAergic neurons; inhibition leads to disinhibition of dopaminergic neurons and subsequent dopamine and glutamate release and AMPA receptor activation (Sanacora et al, 2008).

The antidepressant mechanism of action of ketamine is an area of active research. Hypotheses for the mechanism of action of ketamine involve increased neuroplasticity, increased glutamatergic transmission via AMPA receptors, and increased BDNF (Li et al, 2010; Autry and Monteggia, 2012; Zanos et al, 2018). The general mechanism proposed is that (1) ketamine blocks NMDA receptors on GABAergic neurons leading to (2) a glutamate surge that activates AMPA receptors, resulting in (3) increased BDNF release and mTOR signaling, which increases protein synthesis and AMPA receptor cycling (Li et al, 2010; Abdallah et al, 2015; Suzuki et al, 2017; Kim et al, 2021).

#### 4 Fetal exposure and risks associated with perinatal drug exposure

Ketamine crosses the blood–placenta barrier, as shown in animal and human studies (Ellingson et al, 1977; Cheung and Yew, 2019). Ketamine use is not advised during pregnancy and has been shown to produce adverse effects in offspring in animal studies (Cheung and Yew, 2019). In many animal species, ketamine exposure during pregnancy led to neurodegeneration in fetal brains (Cheung and Yew, 2019). Ketamine effects are dose and time dependent, and fetal exposure and development are key mediators of overall ketamine effects (Cheung and Yew, 2019).

While ketamine during pregnancy is not advised, 2 studies have indicated the effectiveness of ketamine in protecting against postpartum depression. A 2021 study examined the effects of using ketamine to induce anesthesia in women receiving a caesarian section (Alipoor et al, 2021). The authors found that a ketamine dose of 0.5 mg/kg protected against postpartum depression and had no effects on baby health as measured by the Apgar scale (Alipoor et al, 2021). A 2024 randomized, double-blind, placebo-controlled study enrolled 364 mothers and randomized them to receive either a 0.2 mg/kg intravenous infusion of esketamine or placebo over the duration of 40 minutes after childbirth (Wang et al, 2024). The authors found a staggering reduction of a major depressive episode occurring (46/180 participants in the placebo group vs 12/180 participants in the esketamine group). The esketamine-treated group had higher incidence of adverse neuropsychiatric events (40/180 participants in the placebo group vs 82/182 in the esketamine group), for example, dizziness, diplopia, and hallucinations, yet the authors highlighted that these incidents were transient, lasted <1 day, and required no drug intervention. Still, the higher frequency of adverse neuropsychiatric events should be viewed with caution, highlighting the need for more rigorous studies to assess the safety of peripartum ketamine administration. With more information emerging about therapeutic doses and dosing regimens of ketamine, it is not implausible that ketamine may be used in the antenatal or postnatal time points.

## VI Rebranding old drugs for new uses: psychedelics and *κ* opioid receptor antagonists

Many new classes of therapeutics are being developed for antidepressant treatment (see Witkin et al, 2023, for extensive review). Two additional classes of therapeutics that have promising data to support their use as antidepressants are psychedelics and *κ* receptor antagonists. Many studies highlight their potential therapeutic value, which when coupled with their low abuse potential, making these types of drugs highly desirable medications. Psychedelics bring the focus back on the serotonin system. However, instead of working as indirect agonists, for example, like SSRIs, these drugs are agonists at serotonin receptors, particularly 5HT_2A_ receptors. *κ*-Opioid antagonists, as their name suggests, block KORs, potentially reducing dysphoric symptoms.

### A Psychedelics

Psychedelics, colloquially referred to as hallucinogens, are a class of drugs that induce feelings of euphoria, connectedness, and perceptual alterations, with little-to-no abuse potential (Heal et al, 2018). In the 1960s and 1970s, psychedelics were criminalized and still have Drug Enforcement Agency (DEA) schedule I designations, making them difficult to study (Baumeister et al, 2014; Vollenweider and Preller, 2020). Criminalizing psychedelics occurred in response to the antiwar, antiestablishment, hippie movement of the 1970s (Holoyda, 2020). Psychedelics include psilocin, the active compound in psilocybin, *N,N*-dimethyltryptamine, lysergic acid diethylamide, and mescaline (McClure-Begley and Roth, 2022).

While the mechanisms of action of psychedelics differ from SSRIs, both drug classes are agonists at 5HT_2A_ receptors (Baumeister et al, 2014). Recently, Vargas et al (2023) showed that activation of 5HT_2A_ receptors is necessary for the neuroplastic effects of psychedelics. Psychedelics target other receptors as well, including most of the serotonin receptors, all dopamine receptors, and norepinephrine receptors (McClure-Begley and Roth, 2022). Importantly, the psychedelic properties of these drugs are the result of biased agonism, and they display brain region specificity (Vollenweider and Preller, 2020; McClure-Begley and Roth, 2022) because not all drugs that target 5HT_2A_ receptors produce hallucinations (Pottie et al, 2020).

Limited clinical studies have been carried out on psychedelics because of their DEA scheduling. Yet, promising evidence from studies on psilocybin and lysergic acid diethylamide in treating psychiatric disorders such as anorexia nervosa and depression have recently emerged (Tullis, 2021). Long-term effects of microdosing psychedelics are not yet known, nor has safety during pregnancy been systematically assessed (McClure-Begley and Roth, 2022). Thus, pushes for revised scheduling from the DEA and increased research regarding the long-term effects of these drugs are expected to impact their use as antidepressants and anxiolytics. Moreover, efforts to synthesize nonhallucinogenic drugs that produce the neuroplastic effects of psychedelics are on the rise (Cameron et al, 2021). The hypothesized mechanisms of the therapeutic effects of psychedelics are that they enhance synaptic and neuroplasticity, discussed subsequently (McClure-Begley and Roth, 2022).

### B κ-Opioid receptor antagonists

The KORs are important in the stress system, and stress is one of the biggest risk factors for the development of mood and anxiety disorders (Li et al, 2016). In vivo rodent studies highlight the antidepressant effects of KOR antagonists (Lalanne et al, 2014). In the 1980s, U50488, a KOR agonist, was used in clinical trials as a potential therapeutic, but these trials were quickly terminated owing to the dose-dependent dysphoria induced by U50488. Because KOR agonists induce dysphoric effects and KOR antagonists improve mood, KOR antagonists are of particular interest in depressive disorders, particularly, in cases where dysphoria is a core symptom.

**Fig. 2:**
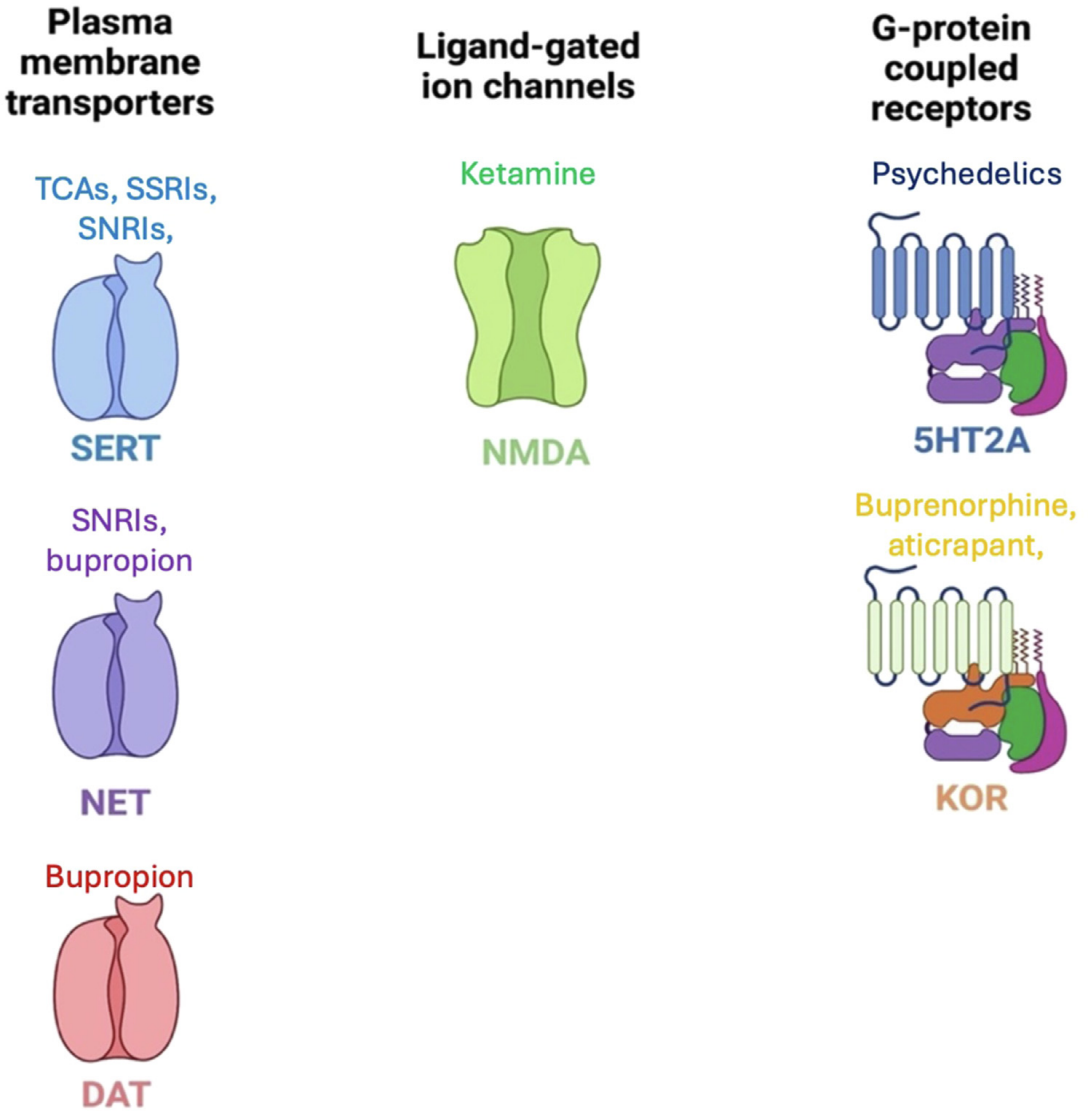
Endogenous targets for pharmacotherapeutics. The SSRIs, SNRIs, and bupropion target plasma membrane transporters. Ketamine targets the ligand-gated ion channel NMDA. Promising therapeutics: psychedelics and KOR antagonists, target 5HT_2A_ and KORs, respectively. All medications described have other molecular targets that may contribute to their effects. DAT, dopamine.

The potential use of KOR antagonists as antidepressants is highlighted in the success of buprenorphine in managing TRD (Karp et al, 2014; Stanciu et al, 2017). Buprenorphine, a drug also used in the treatment of opioid use disorder, is a partial agonist at *μ*-opioid receptors and an antagonist at KORs (Leander, 1988; Lutfy and Cowan, 2004). This dual mechanism of action pointed to *κ* receptors as being important in relieving aversive states associated with withdrawal (Leander, 1988). Based on the success of buprenorphine, the biopharmaceutical company Alkermes developed ALKS 5461, a 1:1 combination of buprenorphine and samidorphan (a potent *μ*-opioid receptor antagonist), for TRD (Li et al, 2016). The FDA gave fast track designation to ALKS 5461 in 2013. However, ALKS 5461 failed to meet primary efficacy endpoints in 2016 and again in 2018 (Peckham et al, 2018). During a similar time, a selective KOR antagonist, aticrapant (LY-2456302 and JNJ-67953964) showed safety, tolerability, and minimal drug interactions (Lowe et al, 2014; Rorick-Kehn et al, 2014). Owing to the limited effectiveness of currently available medications for treating mood and anxiety spectrum disorders and the need for new treatments, the National Institutes for Mental Health initiated a “fast-fail” approach by incorporating a biomarker-based proof of concept in phase IIa clinical trials. Using this strategy, Krystal et al (2020) conducted an 8-week double-blind, placebo-controlled, randomized trial that assessed the effectiveness of aticrapant within 6 academic centers. The KOR antagonism led to positive outcomes in the primary endpoint of changes in ventral striatal activation and improvement in measures of anhedonia, but did not improve broad measures of depression (HAM-D) or anxiety (HAM-A). However, this study inspired further clinical trials, and Schmidt et al (2024) recently reported positive outcomes with significantly reduced depressive symptoms associated with aticrapant as an adjunct therapy, compared with placebo, in patients with major mood disorder who had inadequate response to oral SSRI/SNRI alone. Thus, while KOR antagonists appear promising, further research is needed regarding specific mechanisms of action, including an extensive study of affinity and off-target effects. All medication classes discussed are summarized in Fig. 2 and Table 5.

**Table 5:** Pharmacodynamic summary of antidepressant drug classes Drugs in categories A and B show minimal or no fetal risks in well-controlled clinical and preclinical studies. Drugs in categories C and D show some documented fetal risk associated with their use, but their benefits may outweigh their risks. Drugs in category X should not be used during pregnancy because their benefits are outweighed by their risks.

Medication	Mechanism of Action	FDA Pregnancy Category
SSRIs	Block serotonin reuptake via SERT inhibition; indirect agonists at serotonin receptors	C for all except D for paroxetine
SNRIs	Block serotonin and norepinephrine reuptake via SERT and NET inhibition, respectively; indirect agonists at serotonin and norepinephrine receptors	C for all
Bupropion	Blocks NET and dopamine transporter; antagonist at nicotinic acetylcholine receptors	C
Ketamine	Competitive antagonist at NMDA receptors	NA
Psychedelics	Agonists at 5HT_2A_ receptors	NA
*κ*-Antagonists	Antagonists at *κ* opioid receptors	NA

NA, not applicable.

## VII Beyond molecular targets: shared neurobiological mechanisms of antidepressants

Many of the classical antidepressants, including SSRIs, SNRIs, and TCAs, work at the level of blocking the reuptake of monoamines. As previously described, reuptake inhibition happens rapidly, yet the therapeutic effects of the SSRIs and SNRIs take weeks to months to develop. Thus, while identifying proximal molecular targets are important for understanding antidepressant mechanisms of action, the delayed onset implicates downstream mechanisms of action, that is, compensatory or homeostatic processes, which must be considered to determine more fully how these therapeutics work (Liu et al, 2017).

Antidepressant treatments come in many forms. Pharmacotherapeutics, psychotherapies, and stimulation techniques, for example, ECT, transcranial magnetic stimulation, vagus nerve stimulation, and deep brain stimulation, all provide therapeutic effects. Importantly, while proximal mechanisms differ significantly across treatment modalities and drug classes, all antidepressant treatments cause structural and functional neuroadaptation, processes that underlie neuroplasticity (Pittenger and Duman, 2008; Voss et al, 2017). Important factors that contribute to neuroplasticity include BDNF (and possibly other trophic factors, eg, vascular endothelial growth factor) and synaptogenesis.

### A Brain-derived neurotrophic factor

BDNF is a key mediator of the effects of SSRIs and ketamine, a newly approved antidepressant medication (Bjorkholm and Monteggia, 2016). This growth factor has many roles in the central nervous system, including the regulation of neuronal maturation and synaptic plasticity. Many studies have shown that chronic stress, a key factor for the development of neuropsychiatric disorders, reduces BDNF production in specific brain regions, for example, hippocampus (McEwen, 1999; Vyas et al, 2003; Govindarajan et al, 2006; Martinowich and Lu, 2008; Pittenger and Duman, 2008). Reduced BDNF leads to reduced serotonergic innervation of the hippocampus (Luellen et al, 2007). In contrast, the therapeutic effects of SSRIs depend on increases in hippocampal and cortical BDNF expression (Bjorkholm and Monteggia, 2016). Furthermore, ketamine, which rapidly produces therapeutic effects, transiently increases BDNF in the hippocampus (Abdallah et al, 2015). Thus, BDNF appears to be a key mediator in both the dysfunction produced by stress and the therapeutic effects produced by antidepressants. The effects of BDNF are not limited to pharmacotherapeutics. Both electroconvulsive therapy and other stimulation techniques also result in increases in BDNF production (Bocchio-Chiavetto et al, 2006; Wang et al, 2011).

One of the downstream effects of increased BDNF signaling is increased hippocampal neurogenesis. Postmortem and brain imaging studies have found atrophy and neuronal loss in the prefrontal cortex and hippocampus of depressed or anxious patients (Shah et al, 1998). Studies also show that stress decreases the rates of hippocampal neurogenesis, whereas chronic SSRI use increases neurogenesis (Santarelli et al, 2003; Sachs and Caron, 2014). Taken together, these data suggest that increased BDNF signaling is needed for SSRI efficacy, wherein hippocampal neurogenesis is facilitated (Numakawa et al, 2017). In fact, a current clinical trial is examining the use of BDNF gene therapy for early Alzheimer disease and mild cognitive impairment; the study is set to be completed in 2027 (clinical trial NCT05040217).

### B Synaptogenesis and synaptic strengthening

The therapeutic effects of antidepressants may either be neurogenesis dependent or independent (David et al, 2009). While many studies have shown that neurogenesis is important for SSRI efficacy, the birth of new neurons in adult mammals only occurs in the subventricular zone of the rostral migratory stream and in the subgranular zone of the hippocampal dentate gyrus. Moreover, adult neurogenesis occurs at low rates in primates and decreases with age.

The process by which existing neurons form new synaptic connections is known as synaptogenesis. Neuroplasticity is the subsequent strengthening (or weakening) of existing connections. The number of dendritic spines and synaptic connections is downregulated by stress in the hippocampus, which is mediated, in part, by brain glucocorticoids (McEwen, 1999). In a study, Bessa et al (2009) showed that the therapeutic effects of antidepressants were mediated via neuronal remodeling even when neurogenesis was blocked. Thus, the downstream effects of BDNF on synaptogenesis and synaptic plasticity in regions beyond (and including) the hippocampus, for example, prefrontal cortex and amygdala, are needed for the therapeutic effects of antidepressant pharmacotherapies and other modalities (Pirnia et al, 2016).

### C Global mechanisms

Two global changes that occur in mood and anxiety disorders are increased neuroinflammation and dysfunctional HPA axis signaling (Taylor et al, 2005; Massart et al, 2012). Increased neuroinflammation and dysfunctional HPA axis signaling could be used as biomarkers, which would allow for tangible monitoring of mood and anxiety disorders, although more research is needed (Kennis et al, 2020). During pregnancy, levels of cortisol, BDNF, and neuroinflammatory markers all change, leading to complexities in terms of using these biomarkers perinatally (Mastorakos and Ilias, 2003; Christian et al, 2016; Bränn et al, 2019). Altogether, the etiology of mood and anxiety disorders appears to be multifactorial and presumably attributed to a number of different maladaptive changes that occur in the brain. Future efforts to understand the relationships between these factors will contribute to a holistic understanding of mood and anxiety disorders that may pave the way for new therapeutics.

## VIII Conclusions

Treatment options for women who are experiencing a neuropsychiatric disorder during pregnancy are of utmost importance, especially as the prevalence and incidence of these disorders continue to climb. This is especially relevant considering the recent attacks on access to reproductive health care, including the 2022 *Dobbs v Jackson Women’s Health Organization* decision, which overturned federal protection to abortion access, as well as the increased scrutiny on using medications to end unwanted pregnancy. The United States tragically has the highest rate of maternal mortality among developed countries, especially among Black women, does not guarantee paid family leave at the federal level, and does not have universal childcare (Douthard et al, 2021; Davidson et al, 2024). This leaves pregnant people especially vulnerable because pregnancy can be a highly stressful experience for many individuals, with stress being the biggest known risk factor for developing a mood or anxiety disorder.

Both SSRIs and SNRIs seem to have limited adverse effects on overall fetal health, yet as discussed in this review, SSRIs and SNRIs are not effective for everyone and have a delayed therapeutic onset. With recent advances in psychiatry come novel antidepressants, namely ketamine, which, while not recommended for use during pregnancy, may inspire more efficacious and safe medications in the future. As more knowledge is gained about the anatomical and functional interplay between neurotransmitter systems, therapeutic targets may be identified to address individual variability and to improve personalized medicine.

## Conflict of interest

The authors declare no conflicts of interest.
